# On the Method of Preparing Collodion

**Published:** 1848-08

**Authors:** C. Green

**Affiliations:** Homer, N. Y.


*On the Method of Preparing Collodion.* By C. GREEN, M. D., Homer, N. Y.

The following article by Dr. Green, was received too late for insertion in the department of original communications. As the method of preparing the collodion (which, strangely enough, was not announced by either of the competitors for the honor of the discovery) has not been communicated to our readers, and as some of them may desire to prepare it for themselves, we introduce the article here, rather than defer it for our next No. Dr. G. will please accept our thanks for his valuable contributions.—EDITOR.

I am induced to offer for publication, some remarks on the subject mentioned above, from the consideration that, so far as I have been able to ascertain, no very definite account of the process has been made known to the medical public. A formula for preparing the *cotton* appeared in the Boston Medical and Surgical Journal, but it was very incomplete. In answer to some queries on the subject by a correspondent of that journal, the editor, in the No. for July 19, extracts a formula, with some remarks, from the American Journal of Pharmacy. It appears from those extracts, that the ordinary commercial gun-cotton is *not* soluble in ether. The following is the formula which has been found best to succeed in the preparation of collodion, by the authors of those extracts:—Take of Nitric Acid sp. gr. 1,452; Sulphuric Acid (commercial) each 1 fluid ounce; cleaned and bleached cotton 2 drachms. Thoroughly saturate the cotton with the acids, and macerate for twelve hours. Then wash the cotton, dry it rapidly by artificial heat, in the shade, and dissolve it in one and a half pints of sulphuric ether." The authors of this process (Dr. Parrish and Livermore) remark, that gun cotton, as thus prepared will loose its solubility after being kept a few days, or particularly by being exposed to the rays of the sun; but that which I have prepared, in the manner I am about to relate, is perfectly soluble after being kept several days, and even several weeks. I have found, also, that it is more perfectly soluble when completely and rapidly dried in strong sun light, than when more slowly dried in the shade, by ordinary evaporation. For the proportions of the acid which I

use, I am indebted to the Boston Medical and Surgical Journal. The following is my formula:—Take of pure nitric acid of the shops f. ℨii. commercial sulphuric acid, f. ℨiii. mix and *allow them to cool;* pure cotton (the "drawn cotton" of the factories is the best) 15 grs.; press the cotton into the acids, and see that it is thoroughly saturated with the acids; macerate *thirty minutes.* Then wash the cotton in a glass vessel thoroughly, by means of agitation in repeated waters, and by squeezing, until you can perceive by the taste no remains of acid. Care should be taken not to divide the mass of cotton into many parts, as it will then be more difficult to pick it open equally, and thus fit it for equal and rapid drying, and perfect solubility. After picking the cotton into a light floculent mass, I spread it thinly on a sheet of paper, and expose it to the direct rays of the sun. I suppose that drying by artificial heat would answer the same purpose, but, as yet, I have not tried it. This quantity of cotton may be put into three or four fluid ounces of sulphuric ether, according to the consistence of which you may desire the fluid to be, it being rendered more thin by the addition of ether. The above quantity of cotton with f. ℥iv. will make a solution a little thinner than recently strained honey, and nearly as transparent. It has somewhat the appearance of a thick mucilage of gum arabic. This when dried in thin layers is transparent, and in fact, for some purposes, serves as an excellent varnish. I have observed, that, on standing, the more opaque portion of the solution subsides, and leaves a perfectly transparent stratum on the surface, which, though perfectly adhesive, does not dry as rapidly as the thicker and more opaque portion. It may be shaken before being used.

The *quality* of the gun cotton, and of the solution, will depend much upon the carefulness of the manipulation in the process of preparation. In the first place, if the whole mass of cotton is not perfectly saturated with the acids, a portion, when washed and dried, will not be soluble. In the next place, in the process of maceration and washing, it should be retained in one mass, and not twisted, so that it can be easily separated into flocculae which will favor rapid and perfect drying. It should be entirely freed from acid by agitation in soft-water. The cotton thus prepared will have its natural color, or may be changed to a light yellow, and the color of the solution will be accordingly white or yellow.

In applying it surgically to wounds, the straps should be saturated with it, the edges of the wound held together, and the straps closely applied to the skin until it dries. An additional coating of the collodion may be applied over the straps and around its edges, which will serve to strengthen it. I have used the thin solution with admirable success, as a coating for superficial burns. I should apprehend that it might be of service when applied around the edges of *atonic* ulcers, as it would corrugate the skin, and thereby give support, operating on the principles of Bayton's plan of strapping, or straps saturated with the solution may be used.

In one case of dislocation of the lower end of the ulna from the radius backwards, to which I was called on the 18th day after the accident, and in which I wished to make gentle and continued pressure with a bandage around the lower extremity of the cubital bones, I applied a strap an inch in width, saturated with this solution, and it answered my purpose completely. I was able to make cold application without fear of loosing the strap, or of changing its tenseness. Water dressings with the collodion can be freely used. It will be found a most valuable adjunct in the various departments of surgical practice.

I should have mentioned that before I learned from any source the proportions of the acids to be used, I made a successful experiment by combining sulphuric acid ℥ v., nitric acid, ʒ iii. in which I macerated the cotton and washed as above. It was perfectly soluble. But I have used the Boston proportion, and it is equally good. I should remark, that the cotton thus prepared is not exactly gun cotton. It does not possess its explosive properties.

